# Sigmoid Leiomyoma: An Uncommon Occurrence

**DOI:** 10.7759/cureus.7567

**Published:** 2020-04-06

**Authors:** Arnold N Forlemu, Keng-Yu Chuang

**Affiliations:** 1 Internal Medicine, Creighton University School of Medicine/Maricopa Medical Center, Phoenix, USA; 2 Internal Medicine/Gastroenterology, Valleywise Health, Phoenix, USA; 3 Internal Medicine/Gastroenterology, Creighton University School of Medicine-Phoenix Program, Phoenix, USA

**Keywords:** leiomyoma, sigmoid colon, endoscopic resection, case report

## Abstract

Leiomyomas in the colon are uncommon accounting for a few cases of gastrointestinal smooth muscle tumors. These tumors are usually benign and asymptomatic. They may present with abdominal pain, intestinal obstruction, perforation, and rarely hemorrhagic, especially when the tumor is large. We present the case of a sigmoid leiomyoma in a 60-year-old patient consulting for a positive fecal occult blood test. Colonic leiomyomas should be considered in the differential diagnosis when a polyp is found during routine endoscopic evaluations. This case also highlights the limitations of diagnosing the nature of polyps using endoscopy alone.

## Introduction

Colonic leiomyomas are rare benign tumors that constitute about 3% of gastrointestinal smooth muscle tumors [1]. They often originate either from the muscularis mucosae or the proper muscle itself and frequently occur after the age of 50 years, predominantly in men [2,3]. Colonic leiomyomas are often asymptomatic and are discovered during routine endoscopic evaluations, or they may rarely present with symptoms including abdominal pain and hemorrhage [1]. Complete endoscopic removal of these tumors is difficult because of their submucosal origin. They are however benign tumors with an excellent prognosis and do not recur after removal [4]. We present a case of a 10-mm sessile sigmoid colon polyp that was resected and later found to be a leiomyoma on histology.

## Case presentation

**Figure 1:**
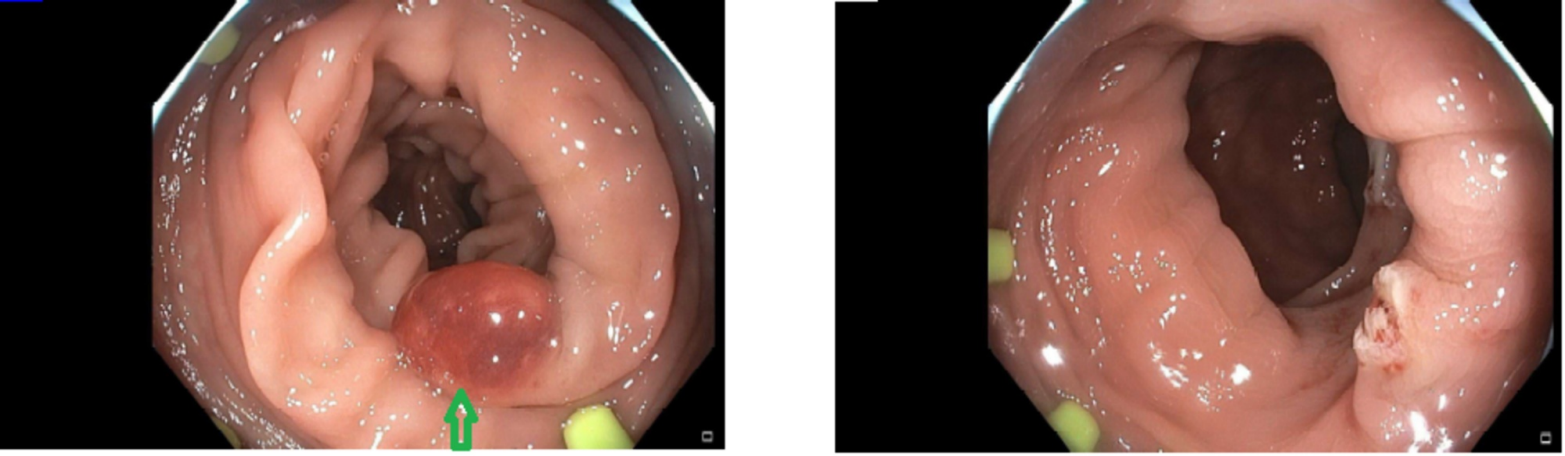
Sigmoid polyp on colonoscopy before and after removal Left: 10-mm red, smooth and round sessile polyp; Right: sigmoid colon after polyp removal

**Figure 2:**
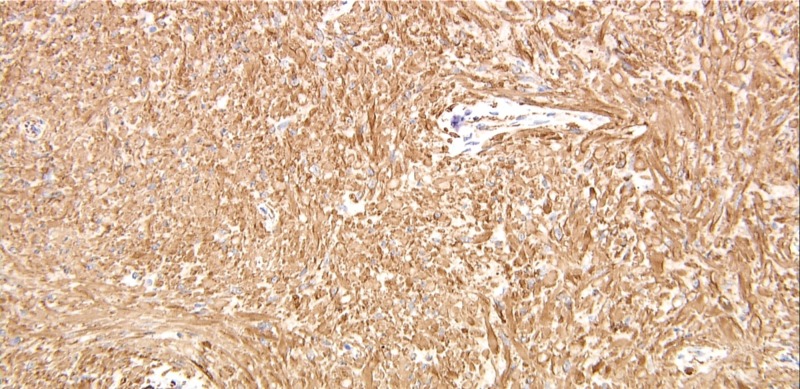
Histologic image of slide showing cells diffusely positive for smooth muscle actin staining Appear brown confirming expression of this smooth muscle marker (magnification: 200x)

A 60-year-old female was seen at our gastroenterology unit for evaluation of a positive fecal occult blood test result. She denied having any gastrointestinal symptoms including abdominal pain, hematemesis, melena, bright red blood per rectum, constipation, dysphagia, weight loss, diarrhea, or heartburn. She had not had age-appropriate colon cancer screening. Physical exam and laboratory testing were non-contributory. Colonoscopy revealed a 10-mm red sessile polyp in the sigmoid colon. The polyp was removed by hot snare polypectomy and sent for histopathology (Figure 1). Histology and immunohistochemistry later revealed the polyp to be a submucosal leiomyoma (Figure 2). The tumor was positive for smooth muscle actin stain but was negative for CD117 ruling out gastrointestinal stromal tumors. Given the beefy red nature of the polyp on endoscopy and no other potential source of bleeding found during endoscopy, the leiomyoma was presumed to be the source of the positive occult blood test.

## Discussion

Colonic leiomyomas or colonic smooth muscle tumors are rare gastrointestinal tumors frequently found around the sigmoid and transverse colon [1]. They can be found in individuals of all ages with an increase in frequency after the age of 60 years [5]. Smooth muscle colonic tumors are often benign; however, some may be locally aggressive or even highly malignant [1]. Even benign looking tumors can metastasize. Hence, tumor size, site, histological appearance, and mitotic count should be considered to predict the malignant potential of these colonic smooth muscle tumors [1]. Most colonic leiomyomas with absent histologic testing are often misdiagnosed as epithelial polyps because they present as solitary sessile intramural or intraluminal polyps [6,7].

Colonic leiomyomas are often asymptomatic and are discovered on routine endoscopic evaluations. They can present with abdominal pain or occult bleeding, as was with our case. They may have a sessile polyp morphology, or present as pedunculated polyps or even as adenomas [2].

Endoscopic removal of colonic leiomyomas is considered an appropriate treatment modality because most of them arise from the muscularis mucosa [7,8]. Several endoscopic techniques, including removal of leiomyomas using cold forceps biopsy, conventional polypectomy, or endoscopic mucosal resection, have been successful without causing bleeding or perforation [7]. Endoscopic snare polypectomy has been successful in completely removing the tumor in most cases [3,8]. However, given the risk of perforation associated with endoscopic removal, lesions to be removed are often lifted using a submucosal injection technique. A positive lift sign would indicate the tumor is superficial enough to be completely resected endoscopically. However, a negative lift sign indicates the tumor is deeper, seated in the muscle itself, and should contraindicate endoscopic removal [7]. Endoscopic mucosal resection often ensures the entire tumor is removed. However, most colonic leiomyomas present as polyps with features suggestive of hyperplastic polyps leading to removal via traditional snare polypectomy. In our case, the polyp was removed via snare polypectomy. Histologic analysis showed a leiomyoma with benign features. The challenge today is to be able to differentiate traditional polyps from leiomyomas endoscopically. Studies are needed to help better suspect leiomyomas endoscopically to ensure the proper technique is used to remove these polyps and save patient cost and stress associated with repeat colonoscopy.

## Conclusions

Leiomyomas are rare in the colon. They are generally benign; however, some smooth muscle colonic tumors may have a potential for malignancy especially after the 60th decade and should be considered in the differential diagnosis when polyps are encountered during routine endoscopic evaluations. Diagnosing colonic leiomyomas via endoscopy alone can be challenging as they are often misdiagnosed as adenomatous polyps. The prognosis of these lesions is however generally excellent, and they often do not recur once removed.
